# Inhibition of CRMP2 Phosphorylation Suppresses Microglia Activation in the Retina and Optic Nerve and Promotes Optic Nerve Regeneration After Optic Nerve Injury

**DOI:** 10.1007/s12017-024-08805-1

**Published:** 2024-09-12

**Authors:** Yuebing Wang, Sayaka Harada, Yoshio Goshima, Toshio Ohshima

**Affiliations:** 1 Department of Life Science and Medical Bioscience, Waseda University, Shinjuku-ku, Tokyo, 162-8480 Japan; 2 Department of Molecular Pharmacology and Neurobiology, Yokohama City University Graduate School of Medicine, Yokohama, 236-0004 Japan; 3 Laboratory for Molecular Brain Science, Department of Life Science and Medical Bioscience, Waseda University, 2-2 Wakamatsu-cho, Shinjuku-ku, Tokyo, 162-8480 Japan

**Keywords:** Collapsin mediator protein 2, Phosphorylation, Microglia, Optic nerve injury

## Abstract

As the primary connection between the eye and brain, the optic nerve plays a pivotal role in visual information transmission. Injuries to the optic nerve can occur for various reasons, including trauma, glaucoma, and neurodegenerative diseases. Retinal ganglion cells (RGCs), a type of neurons that extend axons through the optic nerve, can rapidly respond to injury and initiate cell death. Additionally, following optic nerve injury microglia, which serve as markers of neuroinflammation, transition from a resting state to an activated state. The phosphorylation of collapsin response mediator protein2 (CRMP2) in the semaphorin 3A (Sema3A) signalling pathway affects several processes, including axon guidance and neuron regeneration. In this study, we used an optic nerve crush (ONC) mouse model to investigate the effects of suppressing CRMP2 phosphorylation on microglia activation. We found that CRMP2 phosphorylation inhibitor suppressed RGCs loss and promoted neuronal regeneration following ONC. In addition, CRMP2 S522A mutant (CRMP2 KI) mice exhibited decreased microglial activation in both the retina and optic nerve following ONC. These results suggest that inhibiting the phosphorylation of CRMP2 can alleviate the loss of RGCs and microglial activation after optic nerve injury, providing insight into the development of treatments for optical neuropathies and neurodegenerative diseases.

## Introduction

As our understanding of the central nervous system (CNS) deepens, the challenges associated with mechanistic research on injury and regeneration have correspondingly increased. Neuroinflammation, an enduring research focus, stands at the crossroads of neurodegenerative disease progression and neural repair. As a part of the CNS, the optic nerve transmits visual information from the retina to the brain. Injury to the optic nerve leads to optic neuropathies, which can cause loss of visual function and even blindness. The progressive loss of retinal ganglion cells (RGCs), which are projection neurons in the retina, is linked to several optic neuropathies, including glaucoma and traumatic injury (Levin et al., 1996). Retinal neuroinflammation, defined as the inflammatory response which occurs after optic nerve impairment, is associated with the rapid activation of the retinal microglia (Chang et al., 2009; Rathnasamy et al., 2019). Microglia function as the macrophages of the retina, and are believed to exist in a resting state in the healthy CNS; conversely, under injury conditions, they become sensitive to change, with activated microglia responding to damage, scavenging for pathogens,, aggregates, or debris (Guo et al., 2022). When triggered by neuronal inflammation, injury, and neurodegenerative disorders, microglia exhibit alterations in gene expression and morphology, characterized by an ameboid form (Kettenmann et al., 2011). Previous studies have shown that microglia are activated and migrate following optic nerve injury, triggering them to actively phagocytose cellular debris, including damaged RGCs and their axons (Heuss et al., 2018).

Collapsin response mediator proteins (CRMPs) play diverse roles, including in neuronal development, regeneration, and inflammation (Nakamura et al., 2020). The first discovered member of the CRMP family, CRMP2 (originally named CRMP-62), plays a critical role in the semaphorin 3A (Sema3A) signalling pathway by affecting axonal guidance, dendritic spine development, and synaptic plasticity, primarily through phosphorylation (Goshima et al., 1995; Yamashita et al., 2012). The phosphorylation sites of CRMP2 are predominantly located in its C-terminal region, and cyclin-dependent kinase-5 (Cdk5) is a key kinase acting in CRMP2 phosphorylation. Cdk5 is a serine/threonine kinase that is crucial for the regulation of neuronal development, survival, and apoptosis. It is activated by forming a complex with its neuron-specific activator p35, which phosphorylates CRMP2 at Ser522 (Ohshima et al., 1996). In addition, high calcium levels can convert p35 to p25, thus enhancing Cdk5 activity. CRMP2, in its dephosphorylated form, effectively drives the formation of axonal microtubules, thereby conferring axonal traits to neurons. This mechanism involves the formation of heterotrimers with GTP-tubulin, which ensures the proficient development of axonal microtubules in the nascent axon (Niwa et al., 2017). Upon phosphorylation, CRMP2 exhibits a distinctively opposite effect, leading to growth cone collapse. The phosphorylation triggers conformational changes in the C-terminal tail of CRMP2. This alteration affects both the intramolecular interactions within the CRMP2 tetramer and its interaction with GTP-tubulin. Therefore, phosphorylated CRMP2 is incapable of forming heterotrimers with GTP-tubulin, which in turn impairs its ability to construct and maintain axonal microtubules (Sumi et al., 2018). Based on the previous work on the microglia cellular geometry and neuronal inflammation (Rosito et al., 2023), a hallmark of microglia activation is the remodelling of the microtubule cytoskeleton which highly associated with the function of CRMP2. The phosphorylation of CRMP2 by Sema3A at the Ser522 residue, followed by further phosphorylation by glycogen synthase kinase-3β (GSK3β) at several sites, is associated with significant roles in neuronal processes.

Sema3A, a member of the semaphorin family, has been identified as an extracellular ligand in this phosphorylation pathway that affects growth cone dynamics and axonal elongation (Yamashita et al., 2012). Moreover, Sema3A is known to influence microtubule dynamics and exert regulatory roles in inflammation and immune responses. Recent studies have shown that Sema3A modulates M1-like microglial activation and induces apoptosis in ganglion cells following optic nerve injury (Yun-Jia et al., 2021). Our previous work showed that the suppression of CRMP2 phosphorylation in a CRMP2 S522A mutant (CRMP2 KI) mouse model alleviated RGC death (Brahma et al., 2022). Due to prior research showing the importance of Sema3A-induced CRMP2 phosphorylation and microglial activation after optic nerve injury, we aimed to determine whether CRMP2 phosphorylation affects neuroinflammation after impairment.

Optic nerve crush is a surgical technique widely used in the investigation of optic neuropathies; this technique damages retinal ganglion cell axons, leading to RGC loss (Kondo et al., 2019). Changes of RGC following optic nerve injury are also addressed. A study aimed on relevant properties of RGC apoptosis in the ONC model revealed that p53-induced protein with a death domain can prevent RGC loss via inhibit caspase-2 and tBid activation (Zhang et al., 2019). RGCs experienced the temporal loss after ONC, characterized by two phases: a 65% reduction within the first 7 days, followed by an additional 4% reduction between days 7 and 10 (Sánchez-Migallón et al., 2016). In Rat, apoptosis-related marker, caspase-3 and TUNEL have been verified to participate in the process following ONC (Wu et al., 2014). (-)-Huperzine A (HupA), an alkaloid derived from the herb Huperzia serrata, inhibits CRMP2 phosphorylation at Ser522, thus reducing RGC loss in models of normal-tension glaucoma (Wang et al., 2024). To investigate the role of CRMP2 phosphorylation in Sema3A-induced microglial activation, we employed the optic nerve crush method to create an injury model, and utilized both genetic and chemical approaches to suppress CRMP2 phosphorylation, specifically through the CRMP2 KI model and HupA treatment.

## Materials & Methods

### Experimental Animals

Animal experiments were approved by the Institutional Animal Care and Use Committee of WASEDA University. All mice were housed under a 12-h dark/light cycle, with access to food and water provided ad libitum. Following the results of a previous study (Yamashita et al., 2012), wild-type and CRMP2 KI mice were generated to maintain a hybrid genetic background of the 129 Sv and C57BL/6 J strains. All subjects in the experimental group were male, aged 10–16 weeks at the time of the optic nerve crush (ONC) operation.

### Optic Nerve Crush

Mice were anesthetized by intraperitoneal injection of avertin, and standard ONC was performed as previously described (Cameron et al., 2020). Briefly, all surgical tools were sterilized with 70% ethanol before surgery. Mice were anaesthetizing by Avertin and the anesthesia depth was verified using the hindlimb pinch response test. To expose the eyes, the upper and lower eyelids were gently separated using forceps. A moist cotton swab was used to clear stray hair and excess fluid from around the eye. The conjunctiva was pinched with forceps and incised to create a 1 mm incision. Subsequent enlargement of this incision allowed the removal of the obstructive fat around the optic nerve, avoiding injury to the orbital sinus. Once the optic nerve was fully exposed and visible, it was compressed using self-closing forceps approximately 1–1.5 mm posterior to the globe, for a duration of 5 s.

### Treatment Strategies

HupA (Chemodex, Switzerland) was orally administered to the mice by mixing powder with regular food. The mice were fed a dose of 0.7 mg/kg daily. The duration of HupA administration varied slightly, depending on the experimental design. All groups of mice commenced HupA administration two days prior to ONC, to ensure the rapid action of the active ingredients following ONC.

### Immunostaining

To obtain flat mount retinal samples, mice were intracardially perfused with 4% paraformaldehyde (PFA) in 0.1% PBS (pH 7.4). Eyes were isolated from the eyecup, enucleated, and dissected. Each retina was scored with four evenly spaced slits such that they could be laid flat on the slides. As per a previous retinal sample immunostaining protocol (Wang et al., 2024), following dissection and retinal sampling, retinal samples were incubated in 4% PFA for 24 h for post-fixation. Before treatment with the primary antibody, the retinal samples were incubated in Tris-buffered saline (TBS), methanol, and TBS for 10 min each. Thereafter, the samples were incubated in a blocking solution (3% horse serum in TBS with 0.1% Tween 20 (TBST)) for 1 h. The samples were then incubated with the diluted primary antibody (anti-RBPMS, 1:500, rabbit, PA5-31,231, Invitrogen, CA, USA) overnight at 4 °C. After incubation, samples were washed three times with TBST, for 10 min for each. The samples were then incubated with an Alexa Fluor conjugated secondary antibody for 2 h at room temperature. The samples were then washed twice in TBST for 30 min each, and finally incubated in TBS for 30 min. Immunostained retinal samples were mounted with Fluoromount Aqueous Mounting Medium (F4680; Sigma-Aldrich), and covered with coverslips. Images of the immunostained retinal samples were captured and analyzed using a confocal microscope (FV1000, Olympus) and ImageJ software.

For cryosection, retina and optic nerve samples were placed in PBS at 4 °C (6–12 h), after which the PBS was replaced with 10% sucrose and subsequently 20% sucrose, each time incubated at 4 °C for 12–24 h. The samples were embedded in a solution of Tissue-Tek optimal cutting temperature compound and 20% sucrose at a 2:1 ratio in PBS for the preparation of frozen blocks. Samples were cryo-sectioned longitudinally at 14 μm and mounted on MAS-coated glass slides (Matsunami glass). The immunostaining protocol applied to the optic nerve samples was the same as that used for the retinal samples. The primary antibodies used in this section are Anti-iba1antibody (1:500 rabbit, PAM4131); Biotin anti-Tubulin β (TUBB3) (1:1000, mouse, BioLegend); Anti-GAP43 (Ab-41) antibody (1:1000, rabbit, Sigma-Aldrich, SAB430052); Anti β-actin Monoclonal Antibody (1:1000; Fujifilm Wako). The secondary antibodies were all conjugated with Alexa Flour, in the respective species.

### Western Blot

Mice were euthanized by cervical dislocation at the site of spinal dislocation, and their heads were subsequently dissected. Optic nerve samples spanning from the eye to the optic chiasm were collected for protein extraction for western blotting. These samples were flash frozen in liquid nitrogen and stored at − 80 °C until further use. Protein extraction was carried out by adding 100 μl of Western lysis buffer to the sample tubes, followed by homogenization and centrifugation at 4 °C for one hour. The supernatant was subsequently transferred to new tubes after centrifugation at 12,000 rpm for 15 min at 4 °C. Protein quantification was performed using a BSA (Sigma) calibration standard from the Bio-Rad Protein Assay Kit (Bio-Rad Laboratories), and protein concentrations were determined using GeneQuant 1300 (GE Healthcare). Known protein concentrations were mixed with an appropriate amount of 4 × sample buffer. The protein solution was boiled for 5 min at 90 °C, and then loaded onto a 12.5% polyacrylamide gel for SDS-PAGE analysis. Excel Band 3 Colour Pre-stained Protein Marker (SMOBIO, PM2500-2) was used as a molecular weight marker. Immobilon-P transfer membranes (MILLIPORE) were pre-soaked in 100% methanol (WAKO) and washed with the western blotting buffer. Proteins were transferred from the gel to a membrane at 360 mA for 60 min. Following transfer, membranes were incubated in a blocking buffer at 4 °C for 30 min, and then overnight. Membranes were then incubated in the primary antibodies diluted in blocking buffer for 1 h at room temperature, followed by two washes with 0.05% TBST for 10 min each. The membranes were then incubated in blocking buffer for 10 min at room temperature before reacting with secondary antibodies diluted in blocking buffer for 1–2 h at room temperature. After three washes with 0.05% TBST for 10 min each, the membranes were covered with 1 ml of ECL Plus Western Blotting Detection Reagent (GE Healthcare) for chemiluminescence. Chemiluminescence was detected using a LAS-3000 luminescent image analyzer (FUJIFILM).

### BDA Axonal Tracing

To anterograde trace the axonal regeneration after optic nerve injury, 3 μl of Biotinylated Dextran Amine (BDA-10,000, Thermo Fisher Scientific) was injected intravitreally via a Hamilton syringe (Lazarov, 2013). The mice were intraperitoneally injected with avertin for anesthesia. Mice were dissection four weeks after ONC, and the optic nerve was isolated and post-fixed in 4% PFA overnight. After washing with PBS for 30 min, the optic nerve samples were incubated in 10% and 20% sucrose solution for 12–24 h, after which they were embedded in a 2:1 ratio of Tissue-Tek optimal cutting temperature compound and 20% sucrose in PBS, and frozen by liquid nitrogen. The frozen samples were longitudinally sectioned in a 14 μm slice, and mounted on MAS-coated glass slides (Matsunami glass). The BDA tracing staining method followed the protocol described in our previous study (Kondo et al., 2019). Briefly, the sliced samples were washed in PBS for 30 min and in 0.01% PBST three times for 5 min each. The same blocking solution mentioned above was used to block the samples for 30 min, after which they were incubated in avidin-Alexa568 at 4 °C overnight, blocked from light. After several washes in PBS, the sliced samples were mounted with Fluoromount Aqueous Mounting Medium (F4680; Sigma-Aldrich) and covered with coverslips to avoid air bubbles. Images were captured using an FV1000 confocal microscope (Olympus, Tokyo, Japan). Quantification of the BDA-positive axons extending at distances of 0.5, 1, 1.5, 2, 2.5, and 3 mm from the injury site was performed on a minimum of four nerve sections per mouse. Axons were counted and normalized based on the cross-sectional width of the optic nerve at specified locations as detailed above.

### Statistical Analysis

All data in this study were graphically presented as the mean ± standard error of the mean (SEM). Statistical differences in the two-group analysis were compared using Student’s t-tests with unpaired two-tailed tests and post-hoc tests using Tukey’s multiple-comparison test. For comparisons between more than two groups, statistical differences were calculated using one-way analysis of variance (ANOVA), followed by Tukey’s post-hoc multiple-comparison test, as appropriate to the design. Statistical significance was set at *p* < 0.05 for all analyses, with results indicated by asterisks *p < 0.05, **p < 0.01, ***p < 0.001, ****p < 0.0001. Statistical analyses were performed using GraphPad Prism software version 8.3.0.

## Results

### CRMP2 Phosphorylation Inhibitor Suppressed RGC Loss After Optic Nerve Injury

**Fig. 1:**
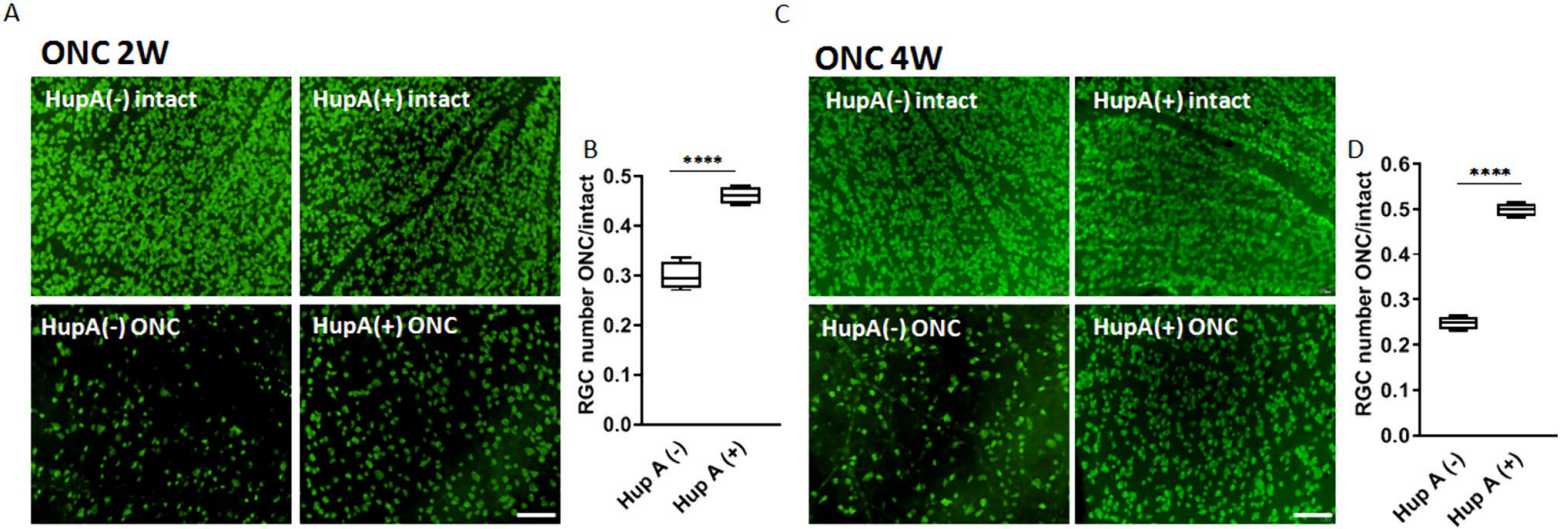
HupA suppresses RGC death after ONC. **A, C** Immunostaining for Anti-RBPMS in flat mount retinas of HupA untreated/treated mice 2 or 4 weeks after ONC. **B, D** Quantification of RGCs 2 or 4 weeks after ONC. Data are presented as the mean ± SEM, statistical comparison was performed using students’ t-tests; Scale bar = 100 μm. (*n* = 4; ****p < 0.0001)

Previous research has suggested that CRMP2 KI, which genetically inhibits CRMP2 phosphorylation, triggers decreased axonal degeneration, improved axonal regeneration, and decreased RGCs loss following optic nerve injury (Kondo et al., 2019). Furthermore, our previous work has shown that HupA exhibits the ability to inhibit CRMP2 phosphorylation at the same site as in the CRMP2 KI mutant (S522)(Y. Wang et al., 2024). To examine the effect of suppressing CRMP2 phosphorylation on the number of RGCs after ONC, anti-RBPMS immunostaining was performed on the retinas of HupA-treated WT mice at two- and four weeks post-ONC. Each mouse underwent ONC on the left eye, while the left right eye was intact as an autologous control. Eight wild-type (WT) mice were randomly divided into HupA-treated and untreated groups. Oral administration of HupA was initiated two days prior to ONC surgery, and was continued until dissection. Whole flat mount retina samples were used in this experiment, with RBPMS-labelled RGCs being counted in twelve regions along the horizontal and vertical directions. The survival rate of RGCs, defined as the ratio of RGCs on the ONC side to those on the intact side in the same mice, served as a metric for quantitative analysis. The results (Fig. 1) showed that noticeable RGC loss was observed in the retina of the ONC side, regardless of the presence or absence of HupA treatment, whereas the intact side maintained a high number of RGCs. This is consistent with previous studies that have shown RGC’s number decrease after optic nerve injury (Berkelaar et al., 1994; Quigley et al., 1995). Comparison between the results of the HupA-treated and untreated groups revealed a significant reduction in the number of RGCs in the eye subjected to the ONC in the treated group. This indicates a mitigating effect of HupA on ONC-triggered RGC loss. Furthermore, upon comparing the outcomes at two and four weeks after ONC, it was evident that the protective effect of HupA on RGCs increased concomitantly with the duration of administration.

### Inhibition of Microglia Activation After Optic Nerve Injury in CRMP2 KI Mice

**Fig. 2:**
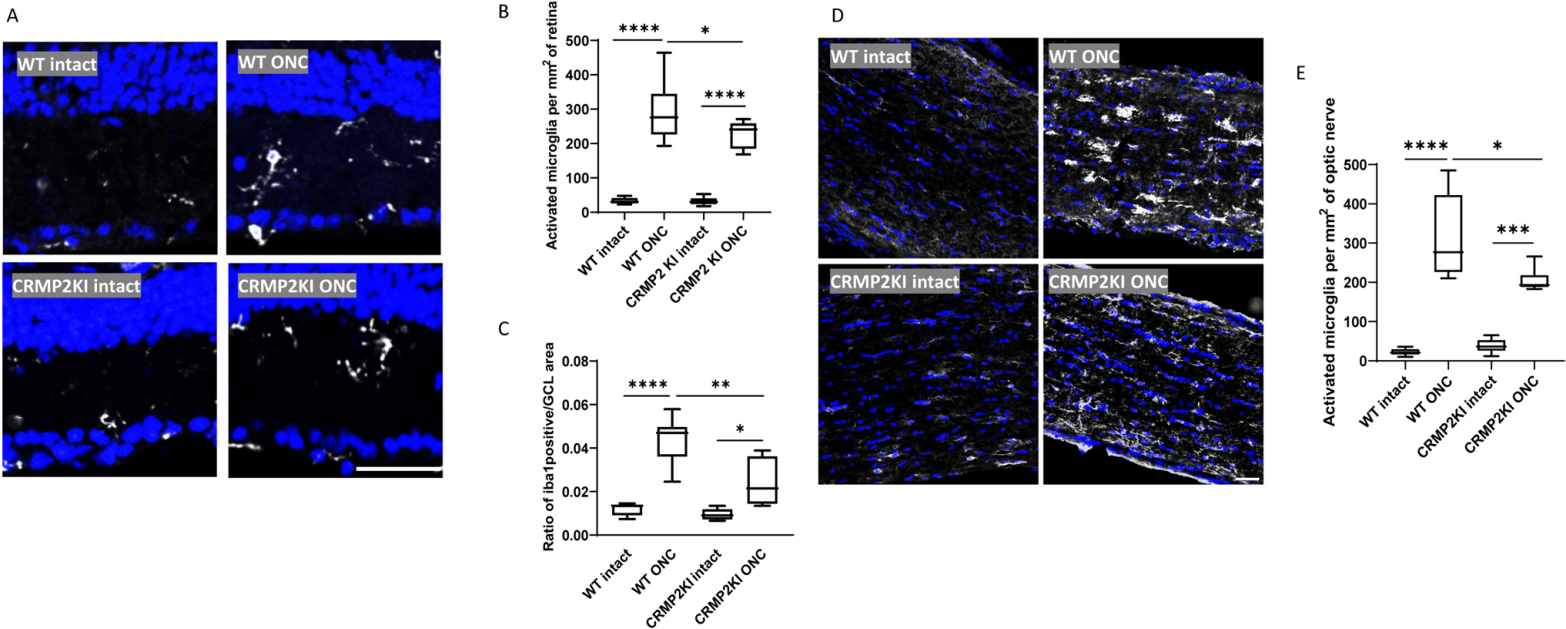
CRMP2 phosphorylation inhibition by CRMP2 KI causes the inhibition of microglia proliferation in the retina and optic nerve. **A** Anti-iba1 and Hoechst Immunostaining on the retina of CRMP2 KI and WT mice 3 days after ONC. **B** Quantification of the number of activated microglia per mm^2^ of retina. **C** The ratio of Iba1 positive signal in the GCL on RGC numbers in each section. **D** Immunostaining for anti-Iba1 and Hoechst in the optic nerves of CRMP2 KI and WT mice 3 days after ONC. **E** Quantification of the number of activated microglia per mm^2^ of optic nerve. Scale bar = 50 μm. Data are presented as the mean ± SEM, statistical comparison was performed using one-way ANOVA, with a Tukey’s multiple comparisons post-hoc test (*n* = 6; *p < 0.05; **p < 0.001; ****p < 0.0001)

The function of the CRMP2 KI mutant in promoting post-ONC neuronal regeneration by inhibiting phosphorylation at S522 has been well established (Kondo et al., 2019). Moreover, an increase in Sema3A expression has been shown to promote the proliferation of M1-like microglia and loss of RGCs after optic nerve injury (Yun-Jia et al., 2021). This section therefore investigates the impact of Sema3A-induced CRMP2 phosphorylation on the neuroinflammatory response characterized by microglial activation after injury. Immunostaining for Iba1 was conducted on both the retina and optic nerves of same-aged WT and CRMP2 KI mice. Six mice from each group were subjected to ONC in the left eye, while the left right eye was left intact as an autologous control. The samples were fixed three days after ONC. For the longitudinally cut retina samples, results were captured at distances ranging from 0.5 to 1.5 mm from the optic disc. The ganglion cell layer (GCL), with a width of 14 μm, was defined based on the DAPI-labelled RGC nuclei. To identify activated microglia, we count cells with an amoeboid morphology, characterized by an enlarged cell body and retracted, thickened processes. In contrast, resting microglia are identified by their ramified morphology, which includes a small cell body and extensive, thin, highly branched processes. The activated microglia number, the ratio of Iba1 positive signals in the GCL were used as metrics for quantitative analysis. For the optic nerve results, four images were uniformly captured from each sample, and Iba1 positive signals were counted as a metric for quantitative analysis. From the staining results of the retina (Fig. 2A), it was evident that microglial activation in the ganglion cell layer (GCL) of the ONC side was significantly higher than that on the intact side. The quantitative analysis demonstrated that the activated microglia on the ONC side were significantly higher than those on the intact side. This aligns with the fact that injury will lead microglia to change from resting to activated. The number of activated microglia on CRMP2 KI mice ONC side was slightly lower than WT ONC, which gives evidence that suppressing phosphorylation of CRMP2 may contribute to inhibiting the microglia activate (Fig. 2B). The ratio of activated microglia to the overall microglial demonstrates that the number of activated microglia is substantially higher on the injured side relative to the intact side (Fig. 2F). Through comprehensive quantitative analysis, it is evident that CRMP2KI mice exhibit a significant reduction in the number of microglia on the ONC side compared to WT mice. This indicates that CRMP2 KI, in which the phosphorylation of CRMP2 at S522 is inhibited, exhibit a certain degree of inhibition on microglial activation. A similar trend was observed in the optic nerve staining results, further corroborating these findings (Fig. 2D, G).

### CRMP2 Inhibitors Alleviate Microglia Proliferation Following Optic Nerve Injury

**Fig. 3:**
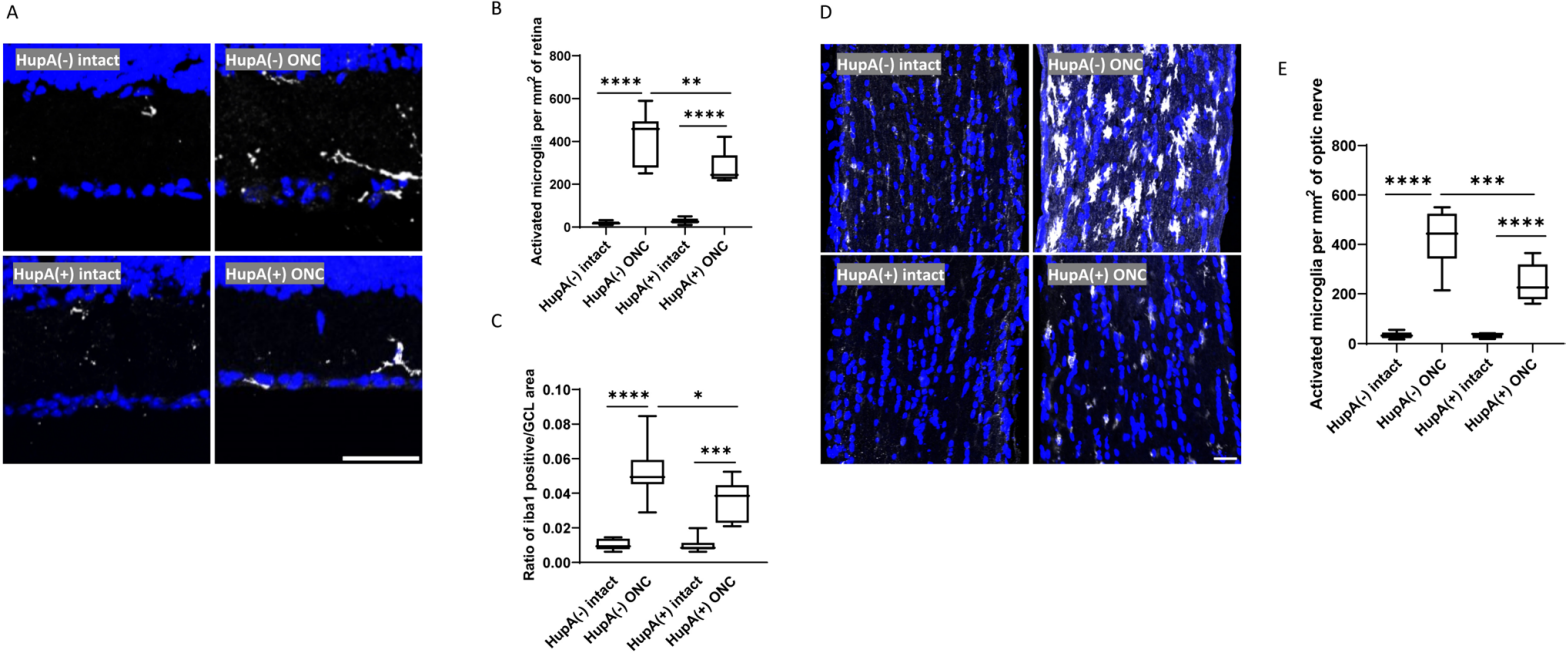
CRMP2 phosphorylation inhibition by HupA causes the inhibition of microglia proliferation in the retina and optic nerve. **A** Immunostaining for Iba1 and Hoechst of HupA untreated/treated retina 3 days after ONC. **B** Quantification of the number of activated microglia per mm^2^ of retina. **C** The ratio of Iba1 positive signal in the GCL on RGC numbers in each section. **D** Immunostaining for Iba1 and Hoechst in the optic nerve of HupA-treated and untreated WT mice 3 days after ONC. **E** Quantification of the number of activated microglia per mm^2^ of optic nerve. Data are presented as the mean ± SEM, statistical comparison was performed using one-way ANOVA, with a Tukey’s multiple comparisons post-hoc test, Scale bar = 50 μm. (*n* = 6, *p < 0.05; ***p < 0.001; ****p < 0.0001)

HupA, a previously validated inhibitor of CRMP2 phosphorylation at S522 (Y. Wang et al., 2024), was considered as another option to suppress CRMP2 phosphorylation in this study, in order to validate our findings in CRMP2 KI mice. Twelve WT mice were randomly assigned to either the HupA-treated or non-treated groups, and HupA administration was initiated two days prior to ONC. Consistent with preceding methodologies, ONC was performed on the left eye, while the right eye served as an autologous control. The retinal samples were fixed three days after ONC. Statistical analysis method was same as CRMP2 KI group. Immunostaining with an anti-Iba1 antibody (Fig. 3A) revealed distinct microglial activation in the GCL on the ONC side compared to that on the intact side. In particular, the quantity of activated microglia on the injured side is significantly higher than on the uninjured side. Quantitative analysis of the positive iba1 signal in the GCL revealed a significant decrease between HupA-treated mice and untreated mice after ONC (Fig. 3C, E). In the optic nerve, HupA-treated mice demonstrated a significant reduction in microglial activation compared to untreated WT mice 3 days after ONC (Fig. 3D, G). A comparison between the HupA-treated and untreated groups indicated a slight reduction in microglial signalling in the GCL on the ONC side following HupA administration, suggesting a suppressive effect of HupA on microglial activation.

### Axon Regeneration in WT Mice Treated with HupA

**Fig. 4:**
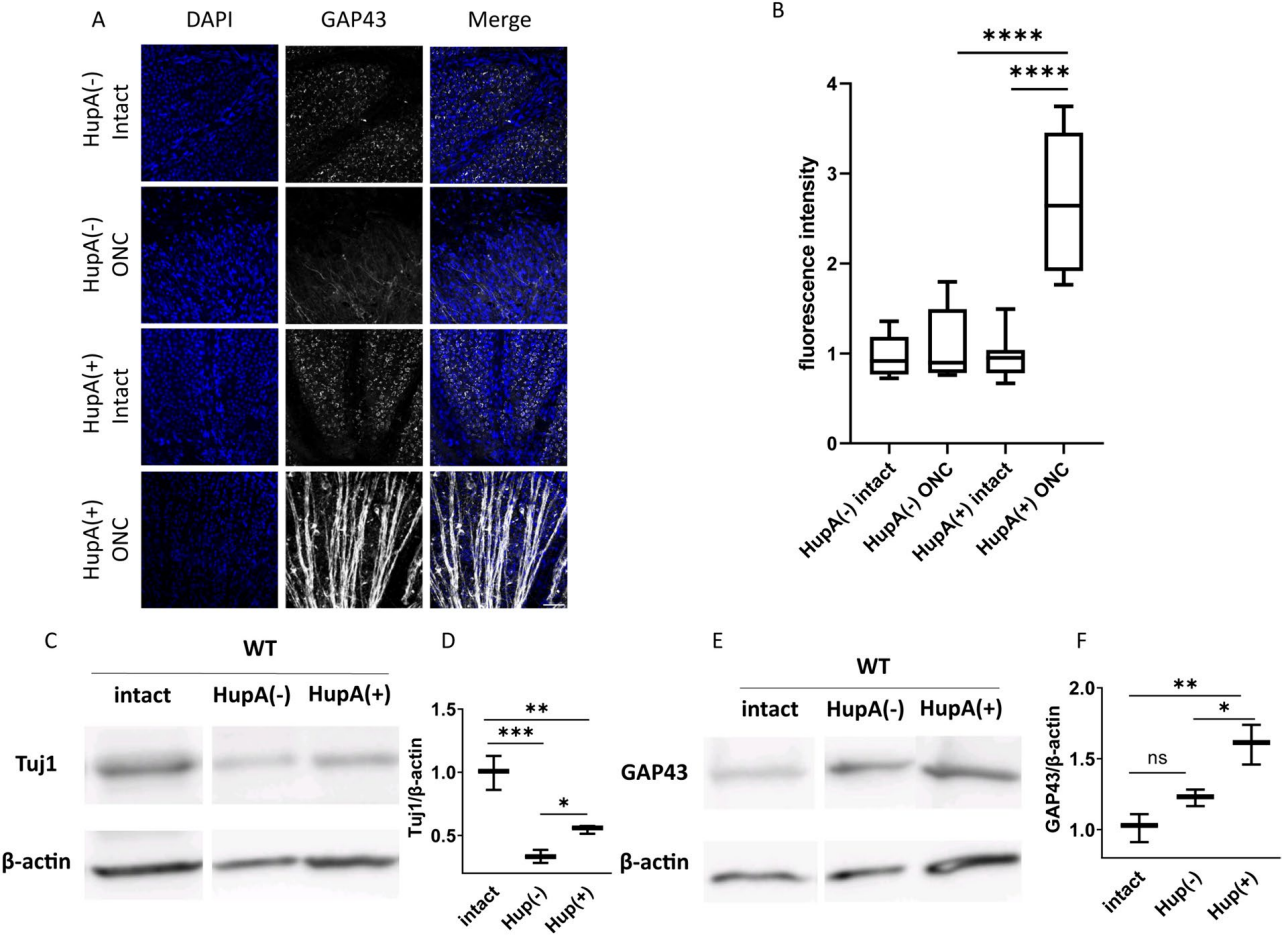
HupA promotes optic nerve regeneration after ONC. **A** Representative images of anti-GAP43 immunostaining on flat mount retina samples from HupA-treated/untreated mice 4 weeks after ONC. **B** Quantification of fluorescence intensity (*n* = 4, Scale bar = 50 μm). **C, E** Western blot of HupA untreated/treated optic nerves. The HupA treatment period lasted 4 weeks. **D, F** Quantification of Tuj1/β-actin and GAP43/β-actin. (*n* = 4, Scale bar = 200 μm) Data are presented as the mean ± SEM, statistical comparison was performed using one-way ANOVA, with a Tukey’s multiple comparisons post-hoc test (*p < 0.05; **p < 0.01; ***p < 0,001; ****p < 0. 0001; ns: no significant)

In addition to microglia labelled with Iba1, this study also examined the alterations in class III β-tubulin marked by Tuj1 and growth-associated protein 43 (GAP43) following ONC injury. Prior research has shown that CRMP2 KI mutants demonstrate increased stabilization of microtubules after spinal cord injury (Nagai et al., 2016). Our previous work has also shown that microtubule stability increases and axon regeneration occurs in CRMP2 KI mice following ONC (Kondo et al., 2019). We performed anti-GAP43 retina immunostaining on HupA-treated WT mice four weeks after ONC, with western blotting of tuj1 and GAP43 four weeks after ONC. GAP-43, a major biomarker associated with growth cones explicitly distributed in axons, is commonly used to evaluate axonal regeneration ability (Kaneda et al., 2008). The anti-GAP43 immunostaining of retina samples was qualified based on relative fluorescence intensity. The results revealed a significant higher GAP43 positive signal in HupA ( +) ONC group compare with HupA (−) ONC group (Fig. 4 A, B). This suggests that HupA promotes axonal regeneration following ONC. Due to the involvement of microtubule dynamics and neuronal regeneration in the validation, we subsequently performed quantitative analysis of the western blots for Tuj1 and GAP43 (Fig. 4 C, D). β-actin served as a housekeeping gene, and the expression level was normalized to the expression of Tuj1 and GAP43 (Tan et al., 2015). Consistent with the immunostaining results, the HupA-treated group exhibited slight but significant upregulation of Tuj1 expression. Both sets of results suggest a protective effect of post-injury HupA treatment against microtubule loss.

### Anterograde Tracing of Optic Nerve Regeneration Improvements

**Fig. 5:**
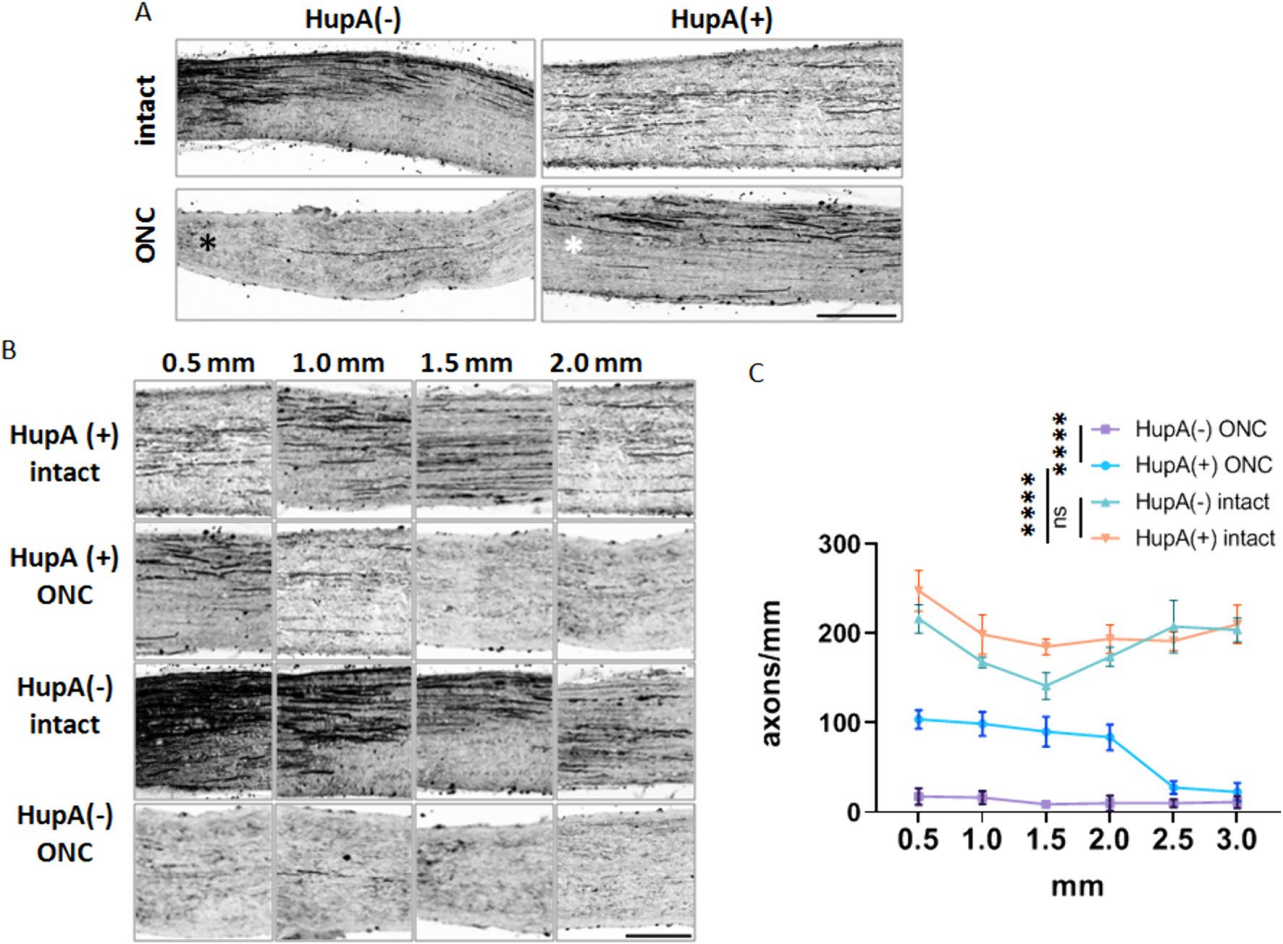
BDA tracing of HupA-treated WT mice 4 weeks after ONC. **A** BDA tracing of HupA untreated/treated optic nerves. The HupA treatment period lasted 4 weeks. Red asterisks mark the injury sites. Scale bar = 500 μm. **B** Magnification of different distances from the crush site. Scale bar = 100 μm. **C** Quantification of regenerating axons at the indicated distances beyond the crush site in optic nerves in HupA untreated/treated mice (*n* = 4) Data are presented as the mean ± SEM, statistical comparison was performed using one-way ANOVA, with a Tukey’s multiple comparisons post-hoc test (****p < 0. 0001; ns: no significant)

BDA, a biotin-conjugated dextran amine, has been widely utilized as a neuroanatomical tracer in many regeneration studies (Au et al., 2022; Yang et al., 2020). Our previous study validated the role of inhibiting CRMP2 phosphorylation at S522 in axonal regeneration following optic nerve injury in a CRMP2 KI model (Kondo et al., 2019). In the current study, we applied the same methodology to investigate the effect of the CRMP2 phosphorylation inhibitor HupA on neuroregeneration four weeks after optic nerve injury. As shown in Fig. 5, the optic nerves of mice receiving continuous HupA administration four weeks after ONC exhibited significantly higher levels of positive axon signals than the non-treated group. This trend aligns with previous findings revealing the efficacy of HupA-mediated inhibition of CRMP2 phosphorylation in promoting post-injury axonal regeneration.

## Discussion

The research direction of this study was intricately connected with those of our previous investigations (Kondo et al., 2019). Previous studies have aimed to elucidate the protective role of inhibiting CRMP2 phosphorylation at S522 using the CRMP2 KI model, whereas this study placed greater emphasis on an alternative phosphorylation inhibition approach, specifically targeting the inhibitor HupA. In the current study, we evaluated the modulatory effect of the CRMP2 phosphorylation inhibitor HupA on RGC death following optic nerve injury. HupA has been used in traditional Chinese medicine for centuries as a remedy for inflammation, schizophrenia, and memory loss (Friedli & Inestrosa, 2021). It is renowned for its ability to antagonize NMDA receptors as an acetylcholinesterase inhibitor (Coleman et al., 2008; Wang et al., 2006). Multiple studies have confirmed the beneficial effects of HupA in the treatment of Alzheimer's disease (AD) (Xu et al., 1995; Yang et al., 2013), while our recent study further demonstrated the inhibitory role of HupA in CRMP2 S522 phosphorylation (Wang et al., 2024), which coincides with the phenotype of the CRMP2 KI mutant. Therefore, the introduction of HupA serves as a complementary approach to inhibit CRMP2 phosphorylation. Moreover, microtubules associated with cytoskeleton dynamics and GAP-43 related to axonal regeneration were examined in the current study to elucidate the axonal regenerative effects of HupA, beyond neuroprotection. As a mature therapeutic agent, the protective effects of HupA against RGC loss after optic nerve injury and its inhibition of neuroinflammatory microglial activation shown in this study provide novel evidence for future clinical research on the treatment of optic nerve lesions. Simultaneously, the regenerative capacity of CRMP2 KI (Kondo et al., 2019) can be partially attributed to its ability to inhibit neuroinflammation triggered by microglial proliferation at the injury site. As a therapeutic agent for AD, Huperzine A is associated with several side effects, including nausea, vomiting, loss of appetite, and increased frequency of bowel movements (Yang et al., 2013). Although HupA has been approved for human use, its efficacy in ameliorating neuroinflammation following optic nerve injury still requires comprehensive clinical trial data for validation. In a meta-analysis study, significant enhancements in cognitive function, as measured by the Mini-Mental State Examination, and improvements in activities of daily living were observed in patients with AD following oral administration of HupA. These effects were noted after a treatment period ranging from 8 to 24 weeks at daily doses of 300 to 500 μg (Wang et al., 2009). The efficacy and optimal dosing of HupA in the treatment of optic neuropathy remain to be elucidated through future clinical trials. HupA has demonstrated therapeutic effects on various neurodegenerative diseases, such as AD and normal-tension glaucoma (NTG) (Wang et al., 2024; Yang et al., 2013). Given the evidence presented in this study regarding HupA’s ability to alleviate neuroinflammation following optic nerve injury, future clinical trials might consider including patients with multiple neurodegenerative conditions and injuries in the inclusion criteria.

The extracellular ligand Sema3A is involved in a variety of biological processes, including neural development and axonal guidance, and plays a crucial role in neuronal positioning and connectivity by aiding in the formation of correct neural networks (Boczek et al., 2014). Microglial proliferation induced by neural verification can be alleviated by inhibiting Sema3A (Yun-Jia et al., 2021). HupA has demonstrated potential therapeutic effects in NTG mouse models by inhibiting the phosphorylation of CRMP2 and modulating M3 mAChR (Wang et al., 2024; Yu et al., 2021). Meanwhile, HupA has been shown to mitigate oxidative stress in NTG models (Wang et al., 2024), similar effects in optic nerve injury models remain to be confirmed in the future study. In this study, we found that CRMP2, a protein in the Sema3a signalling pathway, exhibits an anti-neuroinflammatory effect by inhibiting its phosphorylation. Inhibiting CRMP2 phosphorylation at S522, achieved either through genetic modification of the CRMP2KI mutant or inhibitor administration of HupA, can effectively suppress microglial activation within 3 days post-injury. This finding provides new directions for future studies on other targets in this pathway. It further offers a new option for neuroinflammatory protection against optical neuropathies caused to traumatic optic nerve injury.

To address the limitations of this work, In the present study, the use of a single dose of the CRMP2 phosphorylation inhibitor is a notable one. Using a single dose restricts the ability to determine the full spectrum of the HupA's efficacy and safety. It prevents the identification of an optimal dose and may overlook both sub-therapeutic and toxic effects that could emerge with different dosing regimens. Multiple dose studies are essential to establish a more comprehensive pharmacological profile. Future research should explore varying doses that are tolerable in animal models to elucidate the differences in efficacy among different doses. Moreover, in this study, the administration strategy involved oral HupA administration prior to injury, which does not exclude the possibility of its potential neuroprotective effects before ONC. Future research could explore post-ONC administration to evaluate the therapeutic efficacy of HupA.

In conclusion, this study primarily demonstrates the inhibitory effects on microglial activation following ONC through two approaches: inhibiting CRMP2 S522 phosphorylation via genetic modification and oral administration of HupA. Results in this paper have provided valuable insight into molecular mechanisms of neuroinflammation after the optic nerve injury and the potential treatment of optical neuropathy. Sustained activation of microglia in response to the injury condition are considered a crucial process in pathogenesis. However, the mechanisms underlying the relationship between CRMP2 phosphorylation and neuroinflammation remain unclear. Future studies should address the effects of inhibiting CRMP2 phosphorylation at other sites in the CNS on neuroinflammation, and delve deeper into the molecular mechanisms involved. The potential therapeutic role of inhibiting CRMP2 S522 phosphorylation in optic neuropathy should be supported by further animal experiments and clinical trials. As a potential target for optic nerve injury and other neurodegenerative disease, the small molecule delivery and phosphorylation inhibition of CRMP2 warrant continued in-depth investigation.

## Data Availability

The data that support the findings of this study are available from the corresponding author upon request.

## Abbreviations

RGCs: Retinal ganglion cells
CRMP2: Collapsin response mediator protein 2
Sema3A: Semaphorin 3A
ONC: Optic nerve crush
CRMP2 KI: CRMP2 S522 mutant
CNS: Central nervous system
CRMPs: Collapsin response mediator proteins
Cdk5: Cyclin-dependent kinase-5
GSK3β: Glycogen synthase kinase-3β
HupA: (-)-Huperzine A
PFA: Paraformaldehyde
PBS: Phosphate-buffered saline
TBS: Tris-buffered saline
BDA: Biotinylated dextran amine
GCL: Ganglion cell layer
